# Study of Quantitative Trait Loci (QTLs) Associated with Allelopathic Trait in Rice

**DOI:** 10.3390/genes11050470

**Published:** 2020-04-26

**Authors:** Ill-Min Chung, Tae-Ho Ham, Gi-Won Cho, Soon-Wook Kwon, Yoonjung Lee, Jeonghwan Seo, Yeon-Ju An, So-Yeon Kim, Seung-Hyun Kim, Joohyun Lee

**Affiliations:** 1 Department of Crop Science, Konkuk University, Seoul 05029, Korea; imcim@konkuk.ac.kr (I.-M.C.); lion78@daum.net (T.-H.H.); yoon10.lee@gmail.com (Y.L.); ayj3043@konkko.ac.kr (Y.-J.A.); hellosys1@konkuk.ac.kr (S.-Y.K.); kshkim@konkuk.ac.kr (S.-H.K.); 2 Seed Development Team, Agro Division, Orion Corporation, Gangwon 25323, Korea; gwcho2669@orionworld.com; 3 Department of Plant Bioscience, Pusan National University, Milyang 50463, Korea; swkwon@pusan.ac.kr; 4 Department of Plant Science, Research Institute for Agriculture and Life Sciences and Plant Genomics and Breeding, Seoul National University, Seoul 08826, Korea; jhseo83@snu.ac.kr

**Keywords:** rice, allelopathy, QTL

## Abstract

In rice there are few genetic studies reported for allelopathy traits, which signify the ability of plants to inhibit or stimulate growth of other plants in the environment, by exuding chemicals. QTL analysis for allelopathic traits were conducted with 98 F8 RILs developed from the cross between the high allelopathic parents of ‘Sathi’ and non-allelopathic parents of ‘Nong-an’. The performance of allelopathic traits were evaluated with inhibition rate on root length, shoot length, total length, root weight, shoot weight, and total weight of lettuce as a receiver plant. With 785 polymorphic DNA markers, we constructed a linkage map showing a total of 2489.75 cM genetic length and 3.17 cM of average genetic distance between each adjacent marker. QTL analysis detected on QTL regions on chromosome 8 responsible for the inhibition of shoot length and inhibition of total length. The qISL-8 explained 20.38% of the phenotypic variation for the inhibition on the shoot length. The *qITL-8* explained 14.93% of the phenotypic variation for the inhibition on total length. The physical distance of the detected QTL region was 194 Kbp where 31 genes are located.

## 1. Introduction

Crops are susceptible to attacks from pests and diseases and competition from weeds, which lead to considerable yield losses. Unlike pests and pathogens, which generally invade the crop from external sources, weeds are active within the same cultivation area as the crop and compete for light, nutrients, and water. In the most severe situations, weed competition can lead to complete loss of the crop [1]. Cultivation practices often exacerbate weed germination and development, and weed control is thus essential in most cropping situations. Substantial economic resources are used by farmers for weed-control practices, such as herbicide application [2,3]. Use of allelopathy may allow weeds to be managed in a more cost-effective manner. 

Allelopathy is the ability of plants to inhibit or stimulate growth of plants in the neighboring environment through the activity of exuded bioactive secondary metabolites referred to as allelochemicals [4]. Allelopathic potential in rice was found to be proportional to the amount and type of phytotoxic compounds produced, including phenolic acids and momilactones [5]. Allelochemicals can elicit a wide range of effects, including changes to plant membrane permeability that impact nutrient absorption; suppression of metabolic activities, such as photosynthesis, respiration, and diverse enzyme functions; and disruption of growth and development through inhibition of cell division and elongation and alterations to submicroscopic structures [6].

Plants with allelopathic potential are termed donor plants, whereas plants affected by the allelopathic compounds from the donor plant are referred to as receiver plants. Donor and receiver plants can affect one another through both allelopathy and competition. The combined effect of these two interactions is termed interference [7]. Allelopathic interactions are complex, and it is difficult to exclude the effects of competition and the environment when assessing allelopathic potential. Although field-based screening is an important component of plant-breeding programs, it is almost impossible to distinguish allelopathic potentials from competition under natural field conditions. A range of plant species can be used as receivers in bioassays, to assess allelopathic activity [8] and several considerations, such as susceptibility and genetic uniformity, are important when selecting a receiver species for testing allelopathic potential. Some standard species, such as lettuce (Lactuca sativa), radish (Raphanus sativa), and duckweed (Lemna minor), are recommended for preliminary testing because of their availability and high sensitivity to allelopathic actions [9].

Although many agronomic traits in rice have been studied at the genetic level, relatively few studies have been conducted on the genetic basis of allelopathy. Most published studies to date have involved screening and evaluation of existing plant materials. One quantitative trait loci (QTL) study examined the allelopathic potential of 150 recombinant inbred lines (RILs) generated from a cross between ‘AC1423′, a highly allelopathic rice cultivar, and a minimally allelopathic line, ‘Aus196′. Allelopathic potential was evaluated against the vigorous weed species Echinochloa crus-galli (L.) in laboratory and greenhouse conditions. QTLs linked to allelopathic traits were found on chromosomes 3–10 and 12 [10]. A separate QTL study examined the allelopathic potential of an F2 population derived from ‘PI312777′, a highly allelopathic indica cultivar, and ‘Rexmont’, a minimally allelopathic japonica cultivar. Water-soluble extracts from the F2 seedlings were supplied to lettuce seedlings as receiver plants, and candidate allelopathic QTLs were identified on chromosomes 1, 3, 5, 6, 7, 11, and 12 [11]. 

Recent advances in molecular breeding technologies, such as the development of high-density DNA markers, DNA chips, and next-generation sequencing (NGS), have facilitated the identification and characterization of many genes associated with quantitative traits. However, genetic studies to uncover the basis of allelopathic traits in rice remain in their infancy. This is due to the complications arising from distinguishing allelopathic potential from the effects of competition and the environment, which necessitate performing large numbers of replicated studies in field and greenhouse conditions, as well as in the laboratory. In this study, QTL analysis to identify candidate regions associated with allelopathic traits in rice was conducted in controlled laboratory conditions.

## 2. Materials and Methods

### 2.1. Allelopathic Assay

**Figure 1:**
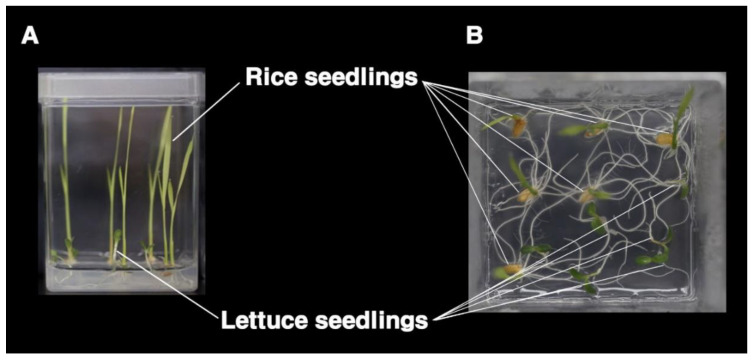
Evacuating method for allelopathic potentials of rice against lettuces seedlings: (**A**) side view of the magenta box where rice seedlings and lettuce seedlings are grown together; (**B**) top view of the magenta box, where rice seedlings and lettuce seedlings are grown together.

Allelopathic potential was assessed by using the equal compartment agar method (ECAM) [12], with minor modifications. A total of 98 F8 RILs were produced by single-seed descent from a cross between ‘Sathi’, an indica cultivar with high allelopathic potential, and ‘Nong-an’, a non-allelopathic Tong-il cultivar [13,14]. Genetically uniform cultivated lettuce ‘Yeolpungjeokchima’ (Lactuca sativa, cv.)—lettuce exhibits high sensitivity to low concentrations of allelopathic chemicals [4]—was used as a receiver species. For ECAM, dehulled rice seeds were sterilized for 15 min, using 2% sodium hypochlorite to prevent fungal and bacterial contamination. Seeds were then rinsed seven times with sterilized distilled water before placing on Whatman No. 5 filter paper (Whatman, Maidstone, England) in a Petri dish (SPL life Sciences, Pocheon, Korea) with 7 mL of sterilized distilled water. Rice and lettuce seeds were germinated in a controlled environment chamber for 3 days, at 28 °C, in the dark. Six germinated rice seeds were transplanted into one side of a Magenta box (SPL Life Sciences, Pocheon, Korea) filled with 30 mL of 0.3% nutrient-free water agar. After 1 week, 6 lettuce seeds were transplanted into the other side of the magenta box (Figure 1). The allelopathic potential of parent rice lines ‘Nong-an’ and ‘Sathi’ and 98 derived F8 RILs were evaluated with ECAM, with lettuce as a receiver plant. Lettuce parameters (root length, root weight, shoot length, shoot weight, total length from base of root to apex of shoot, and total weight) were determined after 1-week co-incubation with rice seedlings. Control lettuce plants were grown in the absence of rice, under the same cultivation conditions. Inhibition rates in lettuce (%) were calculated as follows: [(control − treatment)/control] × 100. Two independent experiments were conducted for each RIL, and thus the average from the total of 12 lettuce plants was used to evaluate each RIL. 

### 2.2. Rice DNA Extraction and High-Throughput SNP Genotyping

DNA was extracted from the parent rice cultivars ‘Nong-an’ and ‘Sathi’ and the 98 F8 RILs, using the CTAB method [15]. The extracted DNA was assessed, using a NanoDrop 2000c spectrophotometer with 230, 260, and 280 nm (Thermo Fisher Scientific, Waltham, MA, USA).

All equipment and resources required for the Axiom 2.0 Assay (Thermo Fisher Scientific, Waltham, MA, USA) with automated target preparation were from the Axiom^®^ 2.0 Assay Automated Workflow (Thermo Fisher Scientific, Waltham, MA, USA). Using the Axiom^®^ 2.0 Reagent Kit, ~200 ng of genomic DNA was amplified and randomly fragmented into 25 to 125 bp fragments and then purified and resuspended. The fragments whose size was confirmed from 25 to 125 bp were denatured and transferred to the hybridization tray in the part of GeneTitan^®^ MC Instrument (Thermo Fisher Scientific, Waltham, MA, USA). The hybridization step followed the GeneTitan^®^ Multichannel Instrument User’s Manual, using the KNU Axiom Oryza 580K Genotyping Array [16] After ligation, the arrays were stained and imaged on the GeneTitan MC Instrument. The image was then analyzed by using Affymetrix^®^ GeneChip^®^ Command Console^®^ Software (Thermo Fisher Scientific, Waltham, MA, USA). Genotype calls were conducted, using Affymetrix-power-tools. BRLMM-P algorithm was applied, which is a model-based approach which performs 1-dimensional clustering by fitting a Gaussian mixture model (BRLMM-P: a Genotype Calling Method for the SNP 5.0 Array [17]). The part of the KNU Axiom Oryza 580K Genotyping Array, the PolyHighResolution chip, which is comprised of 247,578 SNP markers, was used for high-throughput SNP genotyping. The 247,578 SNP markers were designed from the genomic data of 3494 accessions including the Korean rice core set version 2 (KRICE_CORE v2), including wild rice and 3K IRRI world collections [18].

### 2.3. QTL Analysis

A linkage map of the ‘Nong-an’/‘Sathi’ 98 RIL population comprising 785 SNP markers that were polymorphic between parents was constructed by QTL IciMapping 4.1 [19]. For mapping, Kosambi’s function was used, and the Anchor filter option was applied. QTL analysis was conducted by QTL IciMapping 4.1, using inclusive composite interval mapping of additive and epistatic QTLs. Significant LOD threshold value was calculated for each QTL, using 1000 times permutations at *p* = 0.05. The additive effect and phenotypic variation explained by each QTL for allelopathy traits were consequently calculated during QTL analysis, using QTL IciMapping 4.1.

## 3. Results

### 3.1. Screening of Allelopathy in Rice

**Figure 2:**
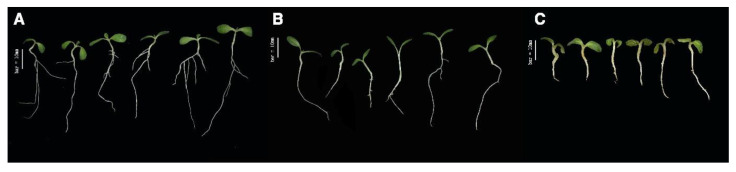
Allelopathic effect on growth inhibition of lettuce: (**A**) lettuces cultivated without rice control; (**B**) lettuces cultivated with rice ‘Nong-an’; and (**C**) lettuces cultivated with rice ‘Sathi’.

**Figure 3:**
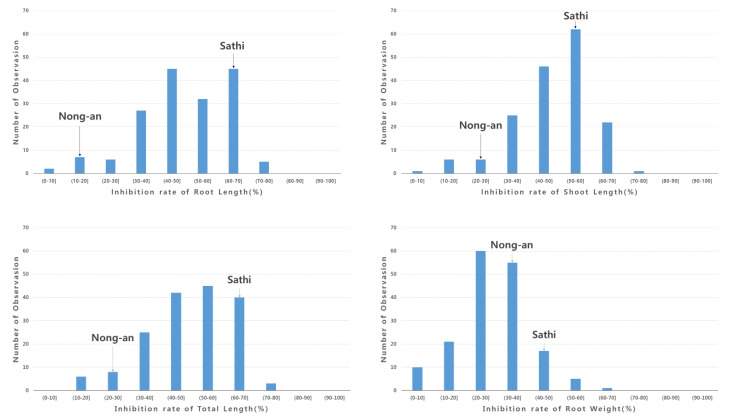

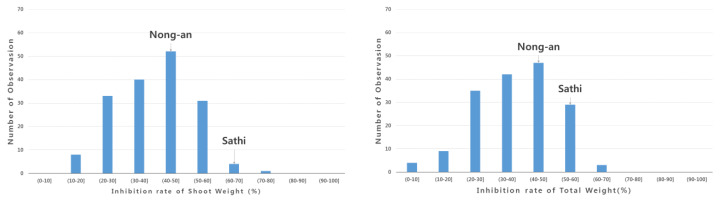
Distribution of growth inhibition rate. (**A**) Inhibition rate of root length, (**B**) inhibition rate of shoot length, (**C**) inhibition rate of total length, (**D**) inhibition rate of root weight, (**E**) inhibition rate of shoot weight, and (**F**) inhibition rate of total weight.

Allelopathic potentials for each RIL were thus represented by the inhibition rates of the six traits. Clear inhibition was detected for all six traits (Figure 2 and Figure 3). Allelopathic differences between the parent cultivars were more apparent for height traits than weight traits. For root length, ‘Sathi’ elicited 62% inhibition, whereas ‘Nong-an’ elicited only 17% inhibition. For shoot length, ‘Sathi’ elicited a 57% inhibition rate compared to 30% with ‘Nong-an’. Overall, total length inhibition was 62% with ‘Sathi’ and 22% with ‘Nong-an’. The highly allelopathic ‘Sathi’ cultivar inhibited both root and shoot length by approximately 60%. By contrast, the non-allelopathic ‘Nong-an’ cultivar elicited stronger inhibition of shoot length (30%) than root length (17%). For root weight, ‘Sathi’ elicited 40% inhibition and ‘Nong-an’ elicited 32% inhibition. For shoot weight, ‘Sathi’ elicited 60% inhibition compared with 42% with ‘Nong-an’. Overall, total weight inhibition was 58% with ‘Sathi’ and 41% with ‘Nong-an’. Although differences in inhibition between the two parent cultivars were relatively small, RILs exhibited large variations in inhibition for all six traits. Transgressive segregation, where RILs exceeded parental phenotypes, was also observed (Figure 3). 

### 3.2. High-Throughput SNP Genotyping and QTL Analysis

**Figure 4:**
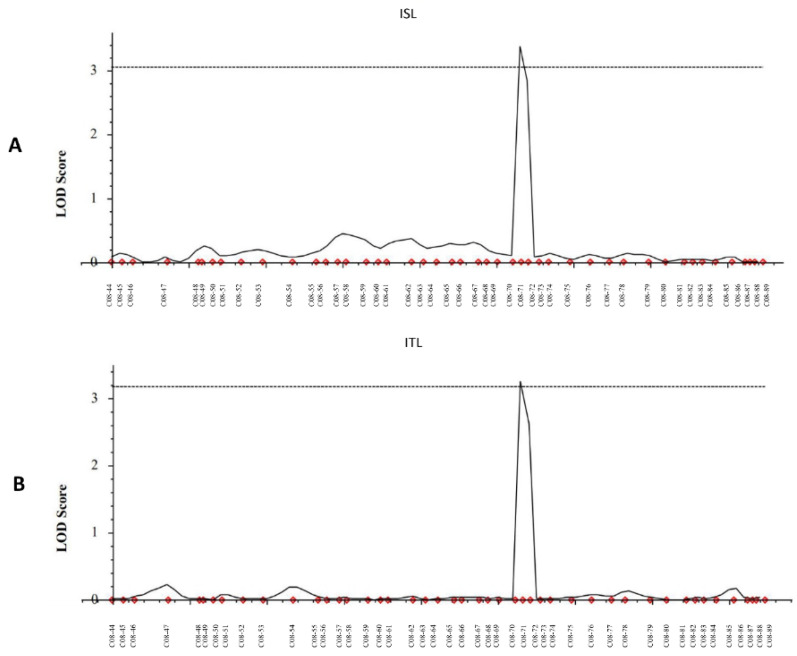
Detected QTLs (qISL and qITL) region associated with inhibition of growth on chromosome 8: (**A**) the LOD score of DNA markers associate with ISL (inhibition rate of shoot length) and position of qISL-8 on chromosome 8; (**B**) the LOD score of DNA markers associate with ITL (inhibition rate of total length) and position of qITL-8 on chromosome 8. Abbreviations are as follows: ISL—inhibition rate of shoot length; ITL—inhibition rate of total length.

For QTL analysis, 785 polymorphic markers between ‘Nong-an’ and ‘Sathi’ were used in constructing a linkage map with 98 RILs (Supplementary Materials Table S1). Among the 247,578 SNP markers used, monomorphic or low-quality markers were eliminated to generate genotypes for 110,770 markers. Additionally, for the generated genotypes for 110,770 markers, binning was carried out for each chromosome, using BIN functionality QTL IciMapping 4.1 to select 2654 markers. Further filtering based on the physical distance (~ 400 kb) was conducted to generate the final 785 markers for the QTL analysis. The markers on the genetic map presented the same ordering presented in the physical map from which the SNP markers were selected, based on the database of IRGSP 1.0. linkage map construct using 98 RIL individuals and 785 markers. Overall, 785 markers were distributed among all 12 rice chromosomes with an average of one marker per 450 kb (Supplementary Materials Figure S1). The number of markers varied from 38 (chromosome 11) to 89 (chromosome 8) with an average of 65.4 per chromosome. The longest length was found in chromosome 1, with 249.5 cM, followed by chromosome 3 with 248.8 cM and by chromosome 12 with 213.2 cM. The linkage map was 2489.75 cM in total length, with the average distance between markers being 3.17 cM. Two main effect-additive QTLs for the allelopathic traits on one chromosome region were detected. One QTL was *qISL-8* (inhibition rate of shoot length), and the other was *qITL-8* (inhibition rate of total length), on chromosome 8, 176.3 cM ~ 177.3 cM (Figure 4). The *qISL-8* showed an LOD value of 3.38, which explained 20.83% phenotypic variance. The qITL-8 showed an LOD value of 3.24, which explained 14.94% phenotypic variance (Table 1). 

**Figure 5:**
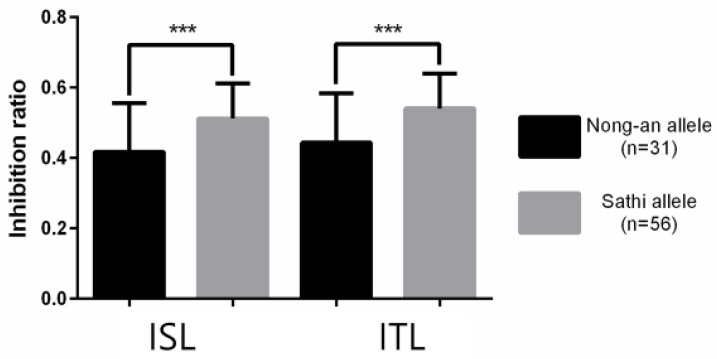
Comparison of inhibition ratio between ‘Nong-an’ and ‘Sathi’ alleles on SNP markers C08-70 and C08-71 for ISL (inhibition rate of shoot length) and ITL (inhibition rate of total length). Significance was determined by *t*-test. *** Indicates significance in 0.001 probability level.

The phenotype differences were compared between ‘Nong-an’ allele and ‘Sathi’ allele on SNP markers C08-70 and C08-71. For the traits of ISL and ITL, the result of the t-test showed significant differences between homozygous alleles of ‘Nong-an’ and ‘Sathi’ (Figure 5).

Two digenic epistatic QTLs for inhibition rate of shoot weight (ISW) and inhibition rate of total weight (ITW) were also identified on identical genomic regions of chromosomes 1 and 8. The two interacting regions for two digenic epistatic QTLs were located in the interval between markers C01-75 and C01-76 on chromosome 1 and the interval between markers C08-42 and C08-43 on chromosome 8. The two digenic epistatic QTLs showed similar phenotypic variance explained (PVE), which are 23.97% for ISW and 23.29% for ITW. Furthermore, additive-by-additive effects of two digenic epistatic QTLs were −7.06 for ISW and −7.31 for ITW (Table 2).

## 4. Discussion

The use of allelopathy for weed control has great potential as a biological control method. Despite this, few genetic studies have examined allelopathy [20] due to the complex challenge of accurately assessing allelopathic interactions in field situations in the presence of natural variability and changing environmental conditions. In this study, an analysis of rice RILs was used to identify QTLs contributing to allelopathic interactions with lettuce, a susceptible receiver plant, in controlled conditions. Six traits were examined, all of which were inhibited in the receiver plant. Inhibition by the different RILs varied widely, and transgressive segregation was observed. Although the cultivation media and growth chamber conditions were well controlled, the conditions in which the receiver plants were cultivated alongside the RILs may produce an inhibitory environment for lettuce growth. Greater inhibition was observed in the non-allelopathic parent (‘Nong-an’) than in lettuce plants grown in the absence of rice. One possibility is that ‘Nong-an’ may exhibit low allelopathy, rather than being strictly non-allelopathic [10]. Alternatively, the receiver plant inhibition may have been at least partly due to competition for space. Although it was not possible to completely eliminate the effects of competition in our experimental design, the inhibitory effect of the highly allelopathic parent ‘Sathi’ was readily apparent, particularly on receiver plant root growth.

Even though we evaluate the allelopathic response with inhibition of weight and length, for the evaluated traits, two main effect additive QTLs for inhibition of root length and inhibition of total length were identified. The locations of both QTLs were identical on the chromosome 8. Because the total length includes the shoot length, this QTL region is mainly for the inhibition of shoot length. The level of explained phenotypic variation for qISL-8 on chromosome 8 was 20.83%. Other previous reports that showed relatively low phenotypic variation explain the value of individual QTL ranging from 5.0% to 11.1%, in general [11]. This relatively low phenotypic variation of values for the individual QTLs is part of the general nature of the allelopathic trait, representing the difficulty in measuring the allelopathic trait at the individual genotype level. In this study, qISL-8 showed a relatively high value of 20.83%, suggesting it is a possible candidate for further study for cloning genes for the allelopathy. The physical distance of the detected QTL region was 194 Kbp where 31 genes are located (Supplementary Materials Table S2). Among them, 12 genes are unknown or hypothetical proteins, and other proteins were reported to be related with auxin response, dehydration, protein kinase, zinc finger protein, chaperone, peroxidase, and isoamylase. Further study for these candidate genes will be conducted, and the development of near-isogenic lines with each QTL are undergoing. The two digenic epistatic QTLs were detected on identical genomic regions for inhibition rate of shoot length (ISL) and inhibition of total weight (ITW). In addition, PVE and the effect of both digenic epistatic QTLs showed ~ 23%. This is possibly the closest related trait between the shoot weight trait and total weight.

## Figures and Tables

**Table 1:** Quantitative trait loci (QTLs) of allelopathy analyzed by inclusive composite interval mapping.

QTL	Trait	Chromosome	Position (cM)	Left Marker	Right Marker	LOD	PVE (%)	Add	Left CI	Right CI
*qISL-8*	ISL	8	177	C08-70	C08-71	3.3848	20.8345	−5.29	176.3	177.3
*qITL-8*	ITL	8	177	C08-70	C08-71	3.2409	14.9362	−5.02	176.3	177.3

Abbreviations are as follows: ISL—inhibition rate of shoot length; ITL— inhibition rate of total length; PVE (%)—phenotypic variance explained; Add—estimated additive effect of Nong—an allele for the QTL; CI—confidence interval calculated by one LOD drop from the estimated QTL position.

**Table 2:** Digenic epistatic QTLs of allelopathy analyzed by inclusive composite interval mapping.

Trait	Chr.	Left Marker	Right Marker	Chr.	Left Marker	Right Marker	LOD	PVE (%)	Add1	Add2	Add by Add
ISW	1	C01-74	C01-75	8	C08-42	C08-43	5.3819	23.9703	−2.39	−3.99	−7.06
ITW	1	C01-74	C01-75	8	C08-42	C08-43	5.2872	23.2899	−2.98	−4.48	−7.31

Abbreviations are as follows: Chr—chromosome; ISW—inhibition rate of shoot weight; ITW—inhibition rate of total weight; PVE(%)—phenotypic variance explained; Add1—estimated additive effect of first QTL; Add2—estimated additive effect of second QTL; Add by Add—additive by additive epistatic effect of QTL at the two scanning points.

## Data Availability

The datasets used and/or analyzed during the current study are available from the corresponding author, upon reasonable request.

## Supplementary Materials

The following are available online at https://www.mdpi.com/2073-4425/11/5/470/s1, Table S1: The 785 SNP markers genotype for 98 RILs. Table S2: The candidate 31 genes located in the detected QTL region on chromosome 8. Figure S1: Linkage map for 12 chromosomes.
