# Supplementary figures and images for: Study of Quantitative Trait Loci (QTLs) Associated with Allelopathic Trait in Rice
Source: Genes (Basel). 2020 Apr 26;11(5):470. doi: 10.3390/genes11050470 (PMC7290725; doi:10.3390/genes11050470)

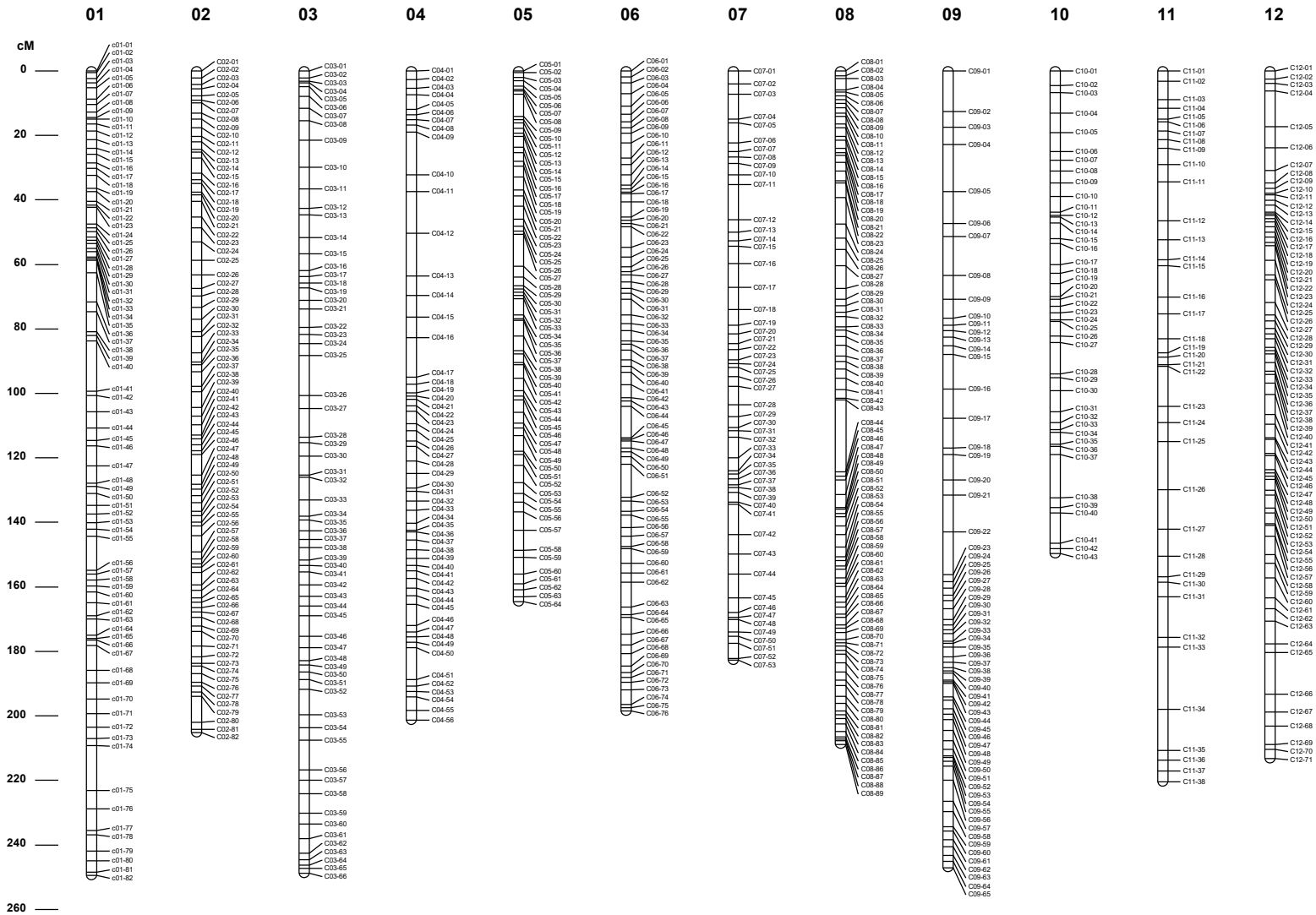

Supplement: Supplementary file 1 [file genes-11-00470-s001.zip › Supplementary_2_Linkage map for 12 chromosomes.pdf]
